# Ultrasonic Spray Pyrolysis Deposited Copper Sulphide Thin Films for Solar Cell Applications

**DOI:** 10.1155/2017/2625132

**Published:** 2017-01-19

**Authors:** Y. E. Firat, H. Yildirim, K. Erturk, A. Peksoz

**Affiliations:** ^1^Physics Department, Sciences and Arts Faculty, Uludag University, Bursa, Turkey; ^2^Physics Department, Sciences and Arts Faculty, Namık Kemal University, Tekirdag, Turkey

## Abstract

Polycrystalline copper sulphide (Cu_*x*_S) thin films were grown by ultrasonic spray pyrolysis method using aqueous solutions of copper chloride and thiourea without any complexing agent at various substrate temperatures of 240, 280, and 320°C. The films were characterized for their structural, optical, and electrical properties by X-ray diffraction (XRD), scanning electron microscopy (SEM), energy dispersive analysis of X-rays (EDAX), atomic force microscopy (AFM), contact angle (CA), optical absorption, and current-voltage (*I-V*) measurements. The XRD analysis showed that the films had single or mixed phase polycrystalline nature with a hexagonal covellite and cubic digenite structure. The crystalline phase of the films changed depending on the substrate temperature. The optical band gaps (*E*_*g*_) of thin films were 2.07 eV (CuS), 2.50 eV (Cu_1.765_S), and 2.28 eV (Cu_1.765_S–Cu_2_S). AFM results indicated that the films had spherical nanosized particles well adhered to the substrate. Contact angle measurements showed that the thin films had hydrophobic nature. Hall effect measurements of all the deposited Cu_*x*_S thin films demonstrated them to be of p-type conductivity, and the current-voltage (*I-V*) dark curves exhibited linear variation.

## 1. Introduction

Copper sulphide (Cu_*x*_S) has attracted a great deal of scientific attention due to the tunable semiconductive properties and approximate metallic behaviors depending on stoichiometry of the mineral phase [1]. In a variety of techniques, it represents a promising p-type semiconductor used in a variety of technologies such as nanoscale electronic devices, cathode material of lithium ion batteries, chemical sensors, and especially photovoltaic cells because of its unique optical and electronic characteristics [2–7]. There are five stable phases of Cu_*x*_S at room temperature which vary according to values of *x* (1 ≤ *x* ≤ 2): chalcocite (Cu_2_S), djurleite (Cu_1.95_S), digenite (Cu_1.8_S), anilite (Cu_1.75_S), and covellite (CuS) [8, 9]. Because of these different phases, the energy band gap of Cu_*x*_S thin films varies between 1.26 and 2.54 eV [10–15].

Many studies have been carried out on Cu_*x*_S films prepared by various techniques such as chemical vapor deposition (CVD) [16], RF sputtering [17], vacuum thermal evaporation [18], spray pyrolysis [19–21], and chemical bath deposition (CBD) [22]. Among these methods, ultrasonic spray pyrolysis (USP) is a suitable method for preparation of Cu_*x*_S thin films [23–25]. The ultrasonic spray pyrolysis technique is a simple coating technology in which an aqueous solution containing compounds in the form of soluble salts of each element is sprayed onto heated substrates. In addition, this process also enables growth of easily nanostructured and high quality thin films and control of film thickness and stoichiometry. In this technique, inexpensive equipment is used, and high quality chemicals and substrates are not necessary compared with the other techniques. The USP is promising due to low cost, easy processability, and the possibility of fabricating large area films with satisfactory structural quality [26]. In USP technique, some parameters such as substrate temperature, molar ratio, type of salt, and pH of the solution change the physical properties of the thin films [27]. Crystalline structure, grain size, optical bad gap, semiconductor type, the ratio of elemental composition, and surface morphology of the deposited thin films can be configured by controlling these USP parameters.

In the present investigation, an attempt was made to deposit polycrystalline Cu_*x*_S thin film of copper sulphide on glass substrates by ultrasonic spray pyrolysis method. Structural, morphological, electrical, and optical properties of copper sulphide thin films were investigated as a function of substrate temperature. Contact angle analysis of the copper sulphide film surfaces was performed depending on the substrate temperature for the first time. We try to explain crystalline phase transitions when the films are deposited at higher temperatures. To our knowledge, such a detailed study on the effect of substrate temperature on the properties of copper sulphide thin films is still lacking.

## 2. Experimental Details

### 2.1. Deposition of Cu_*x*_S Thin Films

In the USP technique, a precursor aerosol is sprayed towards the substrate. The constituents in the precursor droplets interact to form a new chemical compound on the substrate surface. The physical properties of the deposited film vary depending on the composition of solution, spraying rate, substrate temperature, ambient atmosphere, carrier gas, droplet size, and the distance between spray nozzle and substrate. The Cu_*x*_S films were deposited using an ultrasonic spray pyrolysis (USP) technique at different substrate temperatures. Details of the USP technique were described elsewhere [28]. Solution was prepared using a mixture of aqueous solutions of 0.01 M CuCl_2_·2H_2_O and 0.05 M thiourea, CS(NH_2_)_2_. Copper chloride (purity 99.9%) and thiourea (purity 99.0%) were purchased from Sigma-Aldrich. The films were deposited through ultrasonic nozzle onto microscope glass substrates (1 × 2 cm^2^) using air as the carrier gas with pressure of 1 atm. The ultrasonic oscillator frequency was 100 kHz. Deposition time was 20 min and total solution sprayed onto substrates was 100 cc. The solution flow rate was kept at 5 cc/min by a flowmeter. The distance from nozzle to substrate was kept constant at 36 cm. The films were formed at three different substrate temperatures, 240, 280, and 320°C. Substrate temperature was controlled by an iron-constant thermocouple within ±5°C. In this work, the thin films deposited at 240, 280, and 320°C substrate temperatures are called TF1, TF2, and TF3, respectively.

### 2.2. Thin Film Characterization

Characterization of the deposited films was performed using the appropriate techniques. PHE-102 Spectroscopic Ellipsometer (250–2300 nm) was used to measure thicknesses of the films. The X-ray diffraction (XRD) pattern was recorded using a Bruker D8 advanced diffractometer using CuK_*α*_ radiation (*λ* = 1.5406 Å) for 2*θ* values over 20–70°. The surface analysis of the thin films was performed in a Carl Zeiss EVO 40 type SEM (Carl Zeiss NTS Limited Company, Cambridge, UK) operated at 20 kV. The elemental analysis was performed in a Bruker AXS energy dispersive analysis of X-rays (EDAX) operated at 10 keV with an XFlash 4010 detector. Before the analysis, the thin films were fixed on the specimen holder with an aluminum tape and mounted on an aluminum specimen holder. The surface morphology and roughness measurements were studied by atomic force microscope (AFM, Park Systems XE-70). The AFM images were taken in tapping mode with silicon cantilever (NSC16, force constant 40 N/m, resonance frequency 150–190 kHz, and thickness 7.0 *μ*m). The root mean square (RMS) roughness was calculated with the AFM software on images of 4 × 4 *μ*m^2^ scan size. KSV Attention Theta instrument (Hamburg, Germany) was used to determine the contact angles (CA) of the Cu_*x*_S thin films and glass substrate surfaces. Measurements were made in air at room temperature by the sessile drop technique with deionized water. The contact angles of the surfaces were measured via taking 10 separate photos from the different parts of surfaces. The transmittance and absorbance spectra for all the films were carried out using a Shimadzu-SolidSpec-3700 UV-VIS-NIR spectrophotometer. The electrical properties of the films, such as the carrier concentration, Hall mobility, Hall coefficient, and sheet resistance, were characterized by four-point probe and Hall effect measurements (HMS-3000 Manual Ver 3.5.1) at room temperature.

## 3. Results and Discussion

### 3.1. Structural Properties

**Figure 1:**
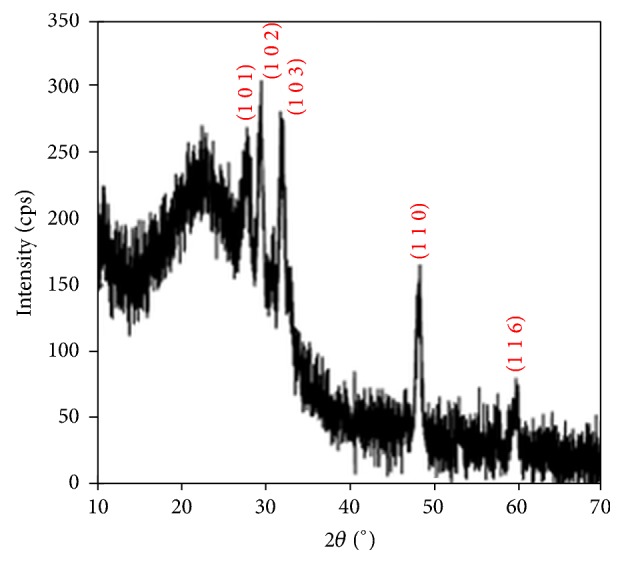
X-ray diffraction patterns of Cu_*x*_S thin film (TF1) deposited at a substrate temperature of 240°C. The diffraction planes were matched with JCPDS card number 6-0464.

**Figure 2:**
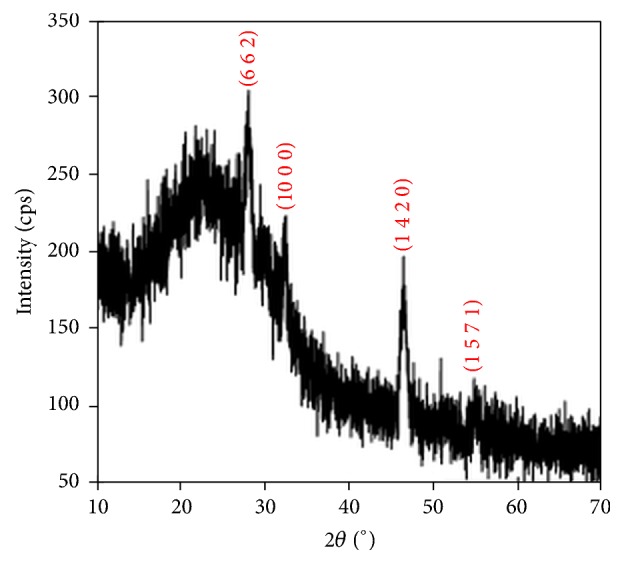
X-ray diffraction patterns of Cu_*x*_S thin film (TF2) deposited at a substrate temperature of 280°C. The diffraction planes were matched with JCPDS card number 23-0960.

**Figure 3:**
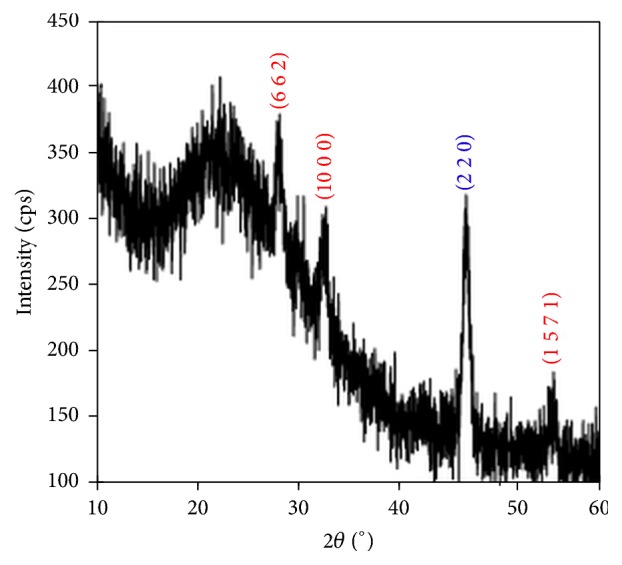
X-ray diffraction patterns of Cu_*x*_S thin film (TF3) deposited at a substrate temperature of 320°C. The diffraction planes were matched with JCPDS card numbers 2-1287 and 23-0960.

The X-ray diffraction patterns (XRD) were recorded for all the films in the range of diffraction angle 2*θ* between 20 and 70°. Figures 1–3 show XRD patterns of the Cu_*x*_S thin films deposited on glass substrates at three different temperature conditions. The X-ray diffraction pattern of these films revealed that these materials were polycrystalline phases: CuS, Cu_1.765_S, and Cu_2_S.

Well-defined $\left(\begin{array}{ccc} 1 & 0 & 1 \end{array}\right)$, $\left(\begin{array}{ccc} 1 & 0 & 2 \end{array}\right)$, $\left(\begin{array}{ccc} 1 & 0 & 3 \end{array}\right)$, $\left(\begin{array}{ccc} 1 & 1 & 0 \end{array}\right)$, and $\left(\begin{array}{ccc} 1 & 1 & 6 \end{array}\right)$ peaks at 2*θ* = 27.74°, 29.48°, 31.76°, 48.24°, and 59.64° are observed in the XRD pattern due to hexagonal covellite phase of CuS (the lattice parameters are as follows: *a* = *b* = 3.792 Å, *c* = 16.344 Å) for the TF1, as shown in Figure 1. The result is in agreement with the data given in the work by Zhu et al. [29] and in standard values (JCPDS 6-0464).  Figure 2 shows the XRD for the TF2. The diffractions peaks at 2*θ* values of 28.16°, 32.26°, 46.46°, and 54.8° can be indexed as $\left(\begin{array}{ccc} 6 & 6 & 2 \end{array}\right)$, $\left(\begin{array}{ccc} 10 & 0 & 0 \end{array}\right)$, (14 2 0), and $\left(\begin{array}{ccc} 15 & 7 & 1 \end{array}\right)$ reflections of the cubic low digenite phase Cu_1.765_S (the lattice parameters are *a* = *b* = *c* = 27.760 Å) for the TF2, which are well matched with the standard values (JCPDS 23-0960). As shown in Figure 3, the TF3 contains the mixed phase of hexagonal chalcocite (Cu_2_S; the lattice parameters are *a* = *b* = *c* = 5.562 Å, JCPDS 2-1287) and cubic low digenite phase (Cu_1.765_S; the lattice parameters are *a* = *b* = *c* = 27.760 Å). The mixture peaks at 2*θ* = 27.92°, 32.44°, and 54.78° are correlated with $\left(\begin{array}{ccc} 6 & 6 & 2 \end{array}\right)$, $\left(\begin{array}{ccc} 10 & 0 & 0 \end{array}\right)$, and $\left(\begin{array}{ccc} 15 & 7 & 1 \end{array}\right)$ planes of Cu_1.765_S and at 2*θ* = 46.62° are correlated with the $\left(\begin{array}{ccc} 2 & 2 & 0 \end{array}\right)$ plane of Cu_2_S.

From the XRD patterns of the Cu_*x*_S films, we conclude that the high substrate temperature leads to a phase transition from Cu_1.765_S to Cu_2_S.

The broadness of the full width at half maximum (FWHM) of the main diffraction peak indicates the formation of nanocrystals [22]. The average crystallite size of the Cu_*x*_S films for the peak with highest intensity can be estimated by using Scherrer's formula [30]:

(1) $$\begin{array}{c} D=\frac{0.9\lambda }{\beta \cos\theta }, \end{array}$$

where *λ* is the wavelength of Cu-K_*α*_ radiation (1.5406 Å), *β* is the broadening of diffraction line measured at half maximum intensity (in radians), and *θ* is the diffraction angle.

The crystallite size was calculated by using the well-known Scherrer formula. The calculated crystallite size varies between 11.92 nm and 9.6 nm for TF1–TF3 materials (Table 1). The results indicate that crystallite size decreases as deposition temperature of the films increases.

### 3.2. Scanning Electron Microscopy and Energy Dispersive X-Rays Analysis

**Figure 4:**
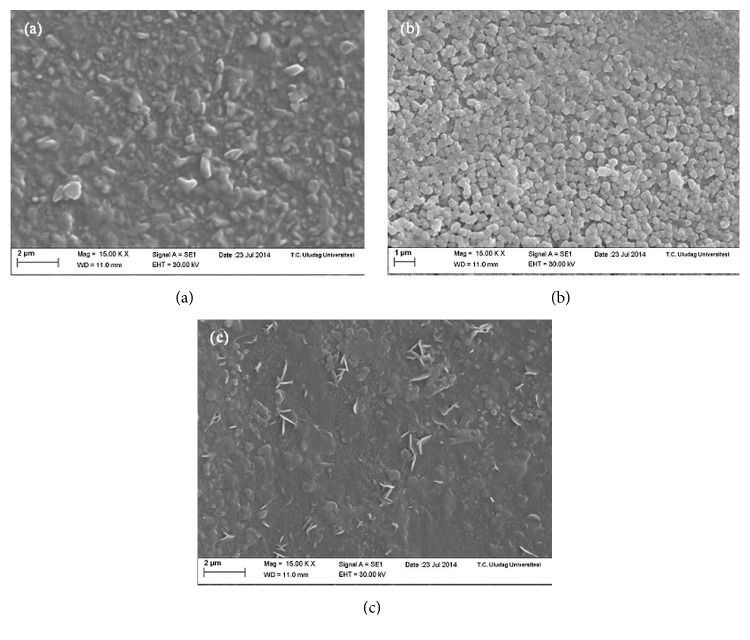
SEM micrographs of (a) TF1, (b) TF2, and (c) TF3.

Figure 4 shows the surface characteristics of the Cu_*x*_S thin films. These films are uniform, dense, and smooth and cover the glass substrate very well. A clear change in the surface formations of the thin films is seen when substrate temperature changes. In the first film, TF1, the surface shows large and small particles in different shapes, and there are no empty spaces between them (Figure 4(a)). The SEM image of TF2 (Figure 4(b)) demonstrates homogeneous distribution of the observed grains with dimensions on the order of 400 nm. There exist a lot of elongated particles with the lengths varying from 900 nm to 100 nm, as well as ellipsoid and circular pieces, on the surface of TF3 (Figure 4(c)).

Energy dispersive analysis of X-rays (EDAX) provides quantitative information about the composition of the thin films. The EDAX results about atomic percentages of Cu and S elements in TF1, TF2, and TF3 thin films are in close agreement with the X-ray diffraction (XRD) results (Table 2).

### 3.3. Atomic Force Microscopy Studies

**Figure 5:**
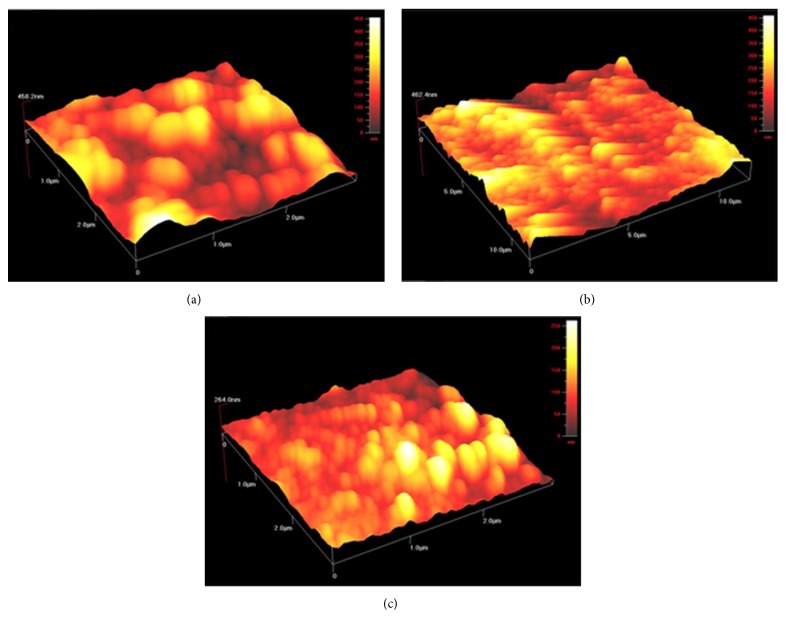
AFM image of (a) TF1, (b) TF2, and (c) TF3.

The control of the surface properties is important in developing for the physical characteristics of the films. Atomic force microscopy (AFM) is used to determine nanoscale surface morphology of the deposited films. These AFM observations allow for controlling surface properties by changing film deposition parameters.  Figure 5 shows three-dimensional (3D) AFM scans of the crystallized Cu_*x*_S thin films grown by USP on glass substrates at 240, 280, and 320°C. The images show that the surface topography is typical, and the morphology of the film indicates roughness and shaped crystallites [7, 31]. The surface rms roughness values of the TF1, TF2, and TF3 thin films are 54.0, 47.2, and 24.3 nm, respectively. The average rms roughness of the films decreases remarkably as the film production temperature increases from 240°C to 320°C. The increase in substrate temperature also brings about an increase in Cu/S ratio of the films. The AFM images of the Cu_*x*_S thin films represent the fact that each of the films is continuous and homogeneous and has no discontinuities or cracked areas (Figure 5).

### 3.4. Contact Angle Measurements

**Figure 6:**
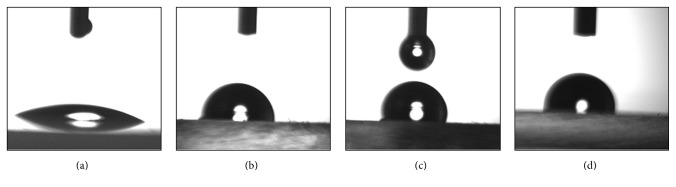
Droplet images of deionized water on (a) glass substrate, (b) TF1, (c) TF2, and (d) TF3.

The contact angle is a macroscopic parameter, which is a consequence of the intermolecular interactions between a liquid and a solid in contact. Contact angle measurements are used to obtain information about the degree of these interactions.  Figure 6 shows the photo-images of water contact angle measurements on the Cu_*x*_S thin films and glass substrate. The water contact angle was equal to 25.76° for untreated glass substrate (Figure 6(a)). The water contact angle measurements on TF1, TF2, and TF3 Cu_*x*_S thin films were found to be 96.60°, 107.83°, and 96.97°, respectively, which show hydrophobic nature of the films (Figures 6(b)–6(d)). The surface porosity of TF1 and TF3 is seen to be similar from the SEM and AFM images (Figures 4 and 5). The contact angles are almost equal to each other for TF1 and TF3 due to this similarity. The surface of TF2 thin film is composed entirely of small and regular grains, while the surface of both TF1 and TF3 has not uniform grain structure. From SEM and AFM observations (Figures 4 and 5), it is concluded that the value of the contact angle is directly correlated with the nanoscale structure of film surfaces. As a result, TF2 has a highest contact angle value of 107.83° due to its different surface topography.

### 3.5. Optical Properties

**Figure 7:**
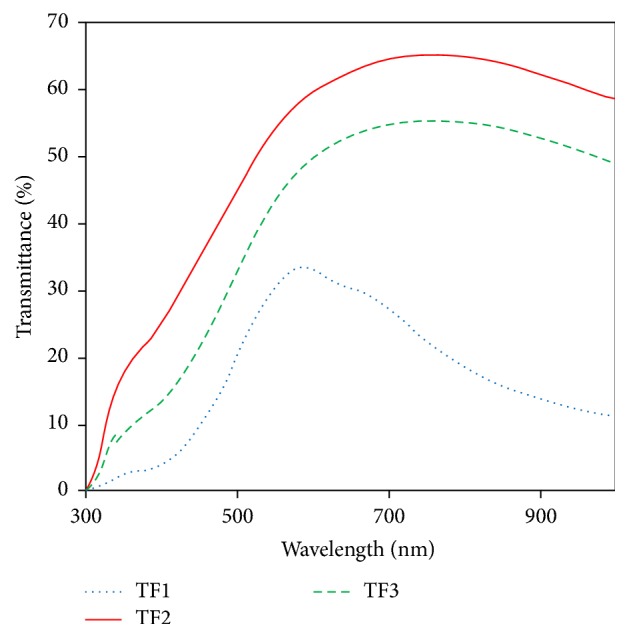
Optical transmission spectra of Cu_*x*_S thin films.

The optical transmittance spectra of Cu_*x*_S films obtained for different deposition temperatures in the range of 200–1100 nm were shown in Figure 7. The transmittance values of the films except for TF1 increase dramatically between 400 and 700 nm wavelength range. The results show that different deposition temperatures are effective on the optical and structural parameters of the films. As seen in XRD pattern of Cu_*x*_S films (Figures 1–3), changing the deposition temperature results in occurrence of different Cu_*x*_S phases. It was found that TF1, TF2, and TF3 films had an average *T* value of ~33%, 65%, and 55% in the visible region, respectively. For TF2 and TF3 films, there is a significant increase in *T*% values depending on the deposition temperature. It is clear that the transmittance of the samples increases as the substrate temperature increases. The thicknesses of TF1, TF2, and TF3 are 160, 380, and 172 nm, respectively. Thus, the film with higher thickness shows an increase in transmittance.

The optical band gaps (*E*_*g*_) for Cu_*x*_S films were also studied and these values were determined using Tauc's law [32, 33]:

(2) $$\begin{array}{c} \alpha h\nu =A{\left(\;h\nu -E_{g}\;\right)}^{m}, \end{array}$$

Where *α* is the absorption coefficient, *hν* is photon energy, *E*_*g*_ is energy band gap, *A* is the edge parameter, and *m* is a constant for a given transition (the value of *m* is 1/2 for direct allowed transitions and 2 for indirect allowed transition).

**Figure 8:**
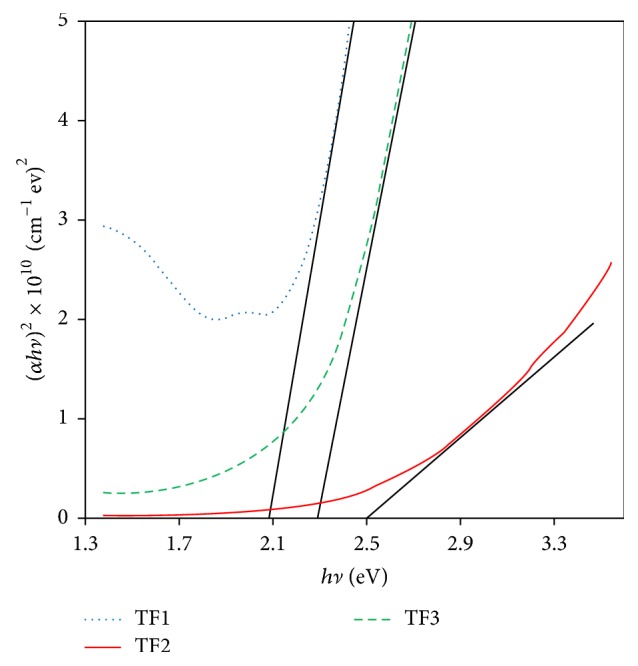
The plot of (*αhν*)^2^ versus photon energy, *hν*, for Cu_*x*_S thin films.

Figure 8 shows plots of square of the product of the optical absorption coefficient (*α*) and photon energy (*hν*) against photon energy. The values of the energy band gaps for the TF1, TF2, and TF3 films are 2.07, 2.50, and 2.28 eV, respectively. It was found that the energy band gaps of the films varied with not only deposition temperature but also Cu_*x*_S phases. XRD pattern given in Figure 1 is evidence that TF1 thin film has CuS single phase, and the calculated energy band gap (*E*_*g*_) using experimental data for TF1 thin film is in agreement with the *E*_*g*_ value reported by Grozdanov and Najdoski [34] and by Zhu et al. [29]. The XRD data shows that TF2 has single phase of Cu_1.765_S. There is no report of *E*_*g*_ values for Cu_1.765_S in the literature, but we have estimated that the *E*_*g*_ value of Cu_1.765_S is between Cu_1.8_S (2.3 eV) [13] and Cu_1.75_S (2.54 eV) [12]. The XRD pattern in Figure 3 demonstrates that TF3 thin film includes both Cu_1.765_S and Cu_2_S phases; that is, this film is a mixture of Cu_1.765_S and Cu_2_S phases. So, there is a small decrease in *E*_*g*_ value compared with TF2 thin film. The reason of this decrease for TF3 thin film may be attributed to having Cu_2_S phase component (1.5 eV) [15] in the mixed phase. Consequently, both optical and structural results found by evaluated experimental data for the films support each other.

### 3.6. Electrical Properties

**Figure 9:**
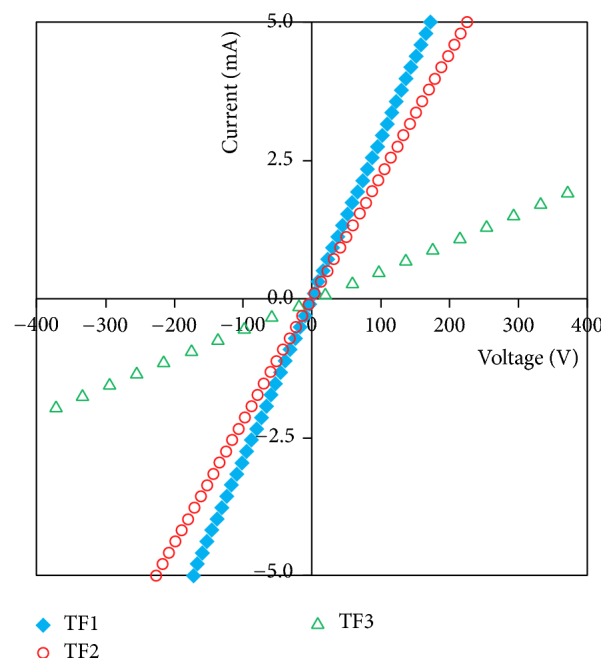
Current-voltage variations for copper sulphide thin films at room temperature.

Hall effect measurements were performed to characterize the electrical properties of the Cu_*x*_S films and all the films showed a p-type semiconductor with hole concentration in the range of (1.45–2.64)·10^21^ cm^−3^ and hole mobility (1.96–11.8)·10^−3^ cm^2^/V·s. Carrier concentration, Hall mobility, Hall coefficient, and sheet resistance values were obtained by four-point probe technique using Hall effect measurement system in a magnetic field strength of 0.556 T and these values were listed in Table 3. As shown in Table 3, all the films present high sheet resistance of (1.25–7.67)·10^3^ (Ω cm)^−1^. It is observed that the carrier mobility values of the films increase as the film crystallite size increases (Tables 1 and 3). It is in agreement with the study reported earlier about Cu_*x*_S films [18]. The current-voltage (*I-V*) curves of the films are obtained in dark (Figure 9). The current increases linearly with the voltage, as seen in Figure 9. Such differences in the* I-V* curves of the films can be correlated with film thickness and/or with amount of defects.

## 4. Conclusions

Copper sulphide thin films were deposited onto glass substrate by spray pyrolysis technique at temperatures 240, 280, and 320°C. It was found that crystalline phase, crystallite size, and surface uniformity of the films were very sensitive to the substrate temperature. The increase of substrate temperature caused crystallite size of the film to decrease by 11.92 nm to 9.60 nm. The film phase was CuS at 240°C and Cu_1.765_S at 280°C. The copper sulphide thin film exhibited polycrystalline structure composed of Cu_1.765_S and Cu_2_S phases when substrate temperature reached 320°C. AFM analysis showed that the surface rms roughness decreased from 54.0 nm to 24.3 nm with the increase of substrate temperature. Sheet resistance of the films increased depending on the increasing substrate temperature. From the optical studies, direct energy band gap values of the films were determined to vary between 2.07 and 2.50 eV. However, any information or data about dependency of optical band gap of the films on substrate temperature could not be found in the literature. In addition to this, optical band gap was found to be dependent on the film thickness and increased from 2.07 to 2.50 eV when film thickness was increased from 160 to 380 nm. The current-voltage variations for Cu_*x*_S thin films were determined to be linear. These films had a p-type semiconductor behavior.

Cu_*x*_S thin films produced in this study can be used in heterojunction solar cells due to their good crystalline structure and energy band gap value matching well solar radiation. Spray pyrolysis deposition represents a low cost technique, allowing the development of large area thin films for solar energy conversion devices.

## Figures and Tables

**Table 1:** Comparison of observed XRD data of thin films with the JCPDS cards. The film thickness, lattice type, crystallite size calculated by Scherrer formula, and contact angle are also summarized.

Thin film	Film thickness (nm)	Observed values		Standard values		hkl	Phase	Lattice	Crystallite size	Contact angle
		2*θ* (°)	d (Å)	2*θ* (°)	d (Å)				D (nm)	(°)
TF1	160	27.74	3.22	27.68	3.22	$\left(\begin{array}{ccc} 1 & 0 & 1 \end{array}\right)$	CuS	Hexagonal	11.92	96.60
		29.48	3.03	29.28	3.05	$\left(\begin{array}{ccc} 1 & 0 & 2 \end{array}\right)$	CuS	Hexagonal		
		31.76	2.80	31.79	2.81	$\left(\begin{array}{ccc} 1 & 0 & 3 \end{array}\right)$	CuS	Hexagonal		
		48.24	1.89	47.94	1.90	$\left(\begin{array}{ccc} 1 & 1 & 0 \end{array}\right)$	CuS	Hexagonal		
		59.64	1.55	59.35	1.56	$\left(\begin{array}{ccc} 1 & 1 & 6 \end{array}\right)$	CuS	Hexagonal		

TF2	380	28.16	3.18	27.95	3.19	$\left(\begin{array}{ccc} 6 & 6 & 2 \end{array}\right)$	Cu_1.765_S	Cubic	10.80	107.83
		32.26	2.76	32.27	2.77	$\left(\begin{array}{ccc} 10 & 0 & 0 \end{array}\right)$	Cu_1.765_S	Cubic		
		46.46	1.95	46.21	1.96	$\left(\begin{array}{ccc} 14 & 2 & 0 \end{array}\right)$	Cu_1.765_S	Cubic		
		54.80	1.67	54.79	1.67	$\left(\begin{array}{ccc} 15 & 7 & 1 \end{array}\right)$	Cu_1.765_S	Cubic		

TF3	172	27.92	3.18	27.95	3.19	$\left(\begin{array}{ccc} 6 & 6 & 2 \end{array}\right)$	Cu_1.765_S	Cubic	9.60	96.97
		32.44	2.76	32.27	2.77	$\left(\begin{array}{ccc} 10 & 0 & 0 \end{array}\right)$	Cu_1.765_S	Cubic		
		46.62	1.95	46.28	1.96	$\left(\begin{array}{ccc} 2 & 2 & 0 \end{array}\right)$	Cu_2_S	Cubic		
		54.78	1.67	54.79	1.67	$\left(\begin{array}{ccc} 15 & 7 & 1 \end{array}\right)$	Cu_1.765_S	Cubic		

**Table 2:** Comparison of EDAX and XRD data.

Thin film	Element	C norm. (wt.%)	C error %	Cu/S ratio (EDAX)	Cu/S ratio (XRD)
TF1	Cu	52.24	0.2	1.094	1.000
	S	47.76	0.2		

TF2	Cu	63.89	0.6	1.769	1.765
	S	36.11	0.5		

TF3	Cu	64.13	0.6	1.788	1.765 and 2.000
	S	35.87	0.4		

**Table 3:** Carrier concentration, Hall mobility, Hall coefficient, sheet resistance, and optical gap values of the Cu_*x*_S thin films.

Cu_*x*_S sample	Carrier concentration (×10^21^ cm^−3^)	Hall mobility (cm^2^/Vs)	Hall coefficient (×10^−4^ cm^3^/C)	Sheet resistance (×10^3^ Ω)	Optical gap (eV)
TF1	2.64	1.18 × 10^−2^	4.42	1.25	2.07
TF2	1.45	8.64 × 10^−3^	1.56	1.29	2.50
TF3	2.41	1.96 × 10^−3^	2.35	7.67	2.28

## References

[1] Orphanou M., Leontidis E., Kyprianidou-Leodidou T., Koutsoukos P., Kyriacou K. C. (2004). Study of copper sulfide crystallization in PEO-SDS solutions. *Langmuir*.

[2] Peng M., Ma L.-L., Zhang Y.-G., Tan M., Wang J.-B., Yu Y. (2009). Controllable synthesis of self-assembled Cu2S nanostructures through a template-free polyol process for the degradation of organic pollutant under visible light. *Materials Research Bulletin*.

[3] Madarász J., Okuya M., Kaneko S. (2001). Preparation of covellite and digenite thin films by an intermittent spray pyrolysis deposition method. *Journal of the European Ceramic Society*.

[4] Lee H., Yoon S. W., Kim E. J., Park J. (2007). In-situ growth of copper sulfide nanocrystals on multiwalled carbon nanotubes and their application as novel solar cell and amperometric glucose sensor materials. *Nano Letters*.

[5] Kim J.-S., Kim D.-Y., Cho G.-B. (2009). The electrochemical properties of copper sulfide as cathode material for rechargeable sodium cell at room temperature. *Journal of Power Sources*.

[6] Sagade A. A., Sharma R., Sulaniya I. (2009). Enhancement in sensitivity of copper sulfide thin film ammonia gas sensor: effect of swift heavy ion irradiation. *Journal of Applied Physics*.

[7] Sharma R., Sagade A. A., Gosavi S. R. (2009). Effect of high electronic energy loss of 100 MeV gold heavy ions in copper chalcogenides (CuX, X = S, Se) at nanoscale: opto-electronic properties study. *Journal of Non-Crystalline Solids*.

[8] Chakrabarti D. J., Laughlin D. E. (1983). The Cu-S (Copper-Sulfur) system. *Bulletin of Alloy Phase Diagrams*.

[9] Goble R. J. (1985). Relationship between crystal structure, bonding and cell dimensions in the copper sulfides. *Canadian Mineralogist*.

[10] Raevskaya A. E., Stroyuk A. L., Kuchmii S. Y., Kryukov A. I. (2004). Catalytic activity of CuS nanoparticles in hydrosulfide ions air oxidation. *Journal of Molecular Catalysis A: Chemical*.

[11] Sagade A. A., Sharma R. (2008). Copper sulphide (Cu_x_S) as an ammonia gas sensor working at room temperature. *Sensors and Actuators, B: Chemical*.

[12] Behboudnia M., Khanbabaee B. (2007). Investigation of nanocrystalline copper sulfide Cu_7_S_4_ fabricated by ultrasonic radiation technique. *Journal of Crystal Growth*.

[13] Naumov A. V., Semenov V. N., Lukin A. N., Goncharov E. G. (2002). Phase composition of copper sulfide films produced from copper salt-thiourea complexes. *Inorganic Materials*.

[14] Rodríguez-Lazcano Y., Martínez H., Calixto-Rodríguez M., Núñez Rodríguez A. (2009). Properties of CuS thin films treated in air plasma. *Thin Solid Films*.

[15] Yu X., An X. (2010). Controllable hydrothermal synthesis of Cu_2_S nanowires on the copper substrate. *Materials Letters*.

[16] Schneider S., Ireland J. R., Hersam M. C., Marks T. J. (2007). Copper(I) tert-butylthiolato clusters as single-source precursors for high-quality chalcocite thin films: film growth and microstructure control. *Chemistry of Materials*.

[17] Yamamoto Y., Yamaguchi T., Tanaka T., Tanahashi N., Yoshida A. (1997). Characterization of CuInS_2_ thin films prepared by sputtering from binary compounds. *Solar Energy Materials and Solar Cells*.

[18] De Carvalho C. N., Parreira P., Lavareda G., Brogueira P., Amaral A. (2013). P-type Cu_x_S thin films: integration in a thin film transistor structure. *Thin Solid Films*.

[19] Isac A., Duta A., Kriza A., Enesca A., Nanu M. (2007). The growth of CuS thin films by Spray Pyrolysis. *Journal of Physics: Conference Series*.

[20] Schneider S., Yang Y., Marks T. J. (2005). Growth of highly oriented chalcocite thin films on glass by aerosol-assisted spray pyrolysis using a new single-source copper thiolate precursor. *Chemistry of Materials*.

[21] Naşcu C., Pop I., Ionescu V., Indrea E., Bratu I. (1997). Spray pyrolysis deposition of CuS thin films. *Materials Letters*.

[22] Mukherjee N., Sinha A., Khan G. G., Chandra D., Bhaumik A., Mondal A. (2011). A study on the structural and mechanical properties of nanocrystalline CuS thin films grown by chemical bath deposition technique. *Materials Research Bulletin*.

[23] Isac L., Duta A., Kriza A., Manolache S., Nanu M. (2007). Copper sulfides obtained by spray pyrolysis—possible absorbers in solid-state solar cells. *Thin Solid Films*.

[24] Kim W.-Y., Palve B. M., Pathan H. M., Joo O.-S. (2011). Spray pyrolytic deposition of polycrystalline Cu_2_S thin films. *Materials Chemistry and Physics*.

[25] Wang S.-Y., Wang W., Lu Z.-H. (2003). Asynchronous-pulse ultrasonic spray pyrolysis deposition of Cu_x_S (x = 1, 2) thin films. *Materials Science and Engineering B*.

[26] Elangovan E., Ramamurthi K. (2005). A study on low cost-high conducting fluorine and antimony-doped tin oxide thin films. *Applied Surface Science*.

[27] Tarwal N. L., Shinde V. V., Kamble A. S. (2011). Photoluminescence and photoelectrochemical properties of nanocrystalline ZnO thin films synthesized by spray pyrolysis technique. *Applied Surface Science*.

[28] Atay F., Bilgin V., Akyuz I., Kose S. (2003). The effect of In doping on some physical properties of CdS films. *Materials Science in Semiconductor Processing*.

[29] Zhu L., Xie Y., Zheng X., Liu X., Zhou G. (2004). Fabrication of novel urchin-like architecture and snowflake-like pattern CuS. *Journal of Crystal Growth*.

[30] Cullity B. D. (1956). *Elements of X-Ray Diffraction*.

[31] Isac L., Andronic L., Enesca A., Duta A. (2013). Copper sulfide films obtained by spray pyrolysis for dyes photodegradation under visible light irradiation. *Journal of Photochemistry and Photobiology A: Chemistry*.

[32] Ubale A. U., Choudhari D. M., Kantale J. S. (2011). Synthesis of nanostructured Cu_*x*_S thin films by chemical route at room temperature and investigation of their size dependent physical properties. *Journal of Alloys and Compounds*.

[33] Pankove J. I. (1971). *Optical Processes in Semiconductors*.

[34] Grozdanov I., Najdoski M. (1995). Optical and electrical properties of copper sulfide films of variable composition. *Journal of Solid State Chemistry*.

